# Spontaneous Regression of Ovarian Carcinoma After Septic Peritonitis; A Unique Case Report

**DOI:** 10.3389/fonc.2018.00562

**Published:** 2018-11-29

**Authors:** Thijs Roelofsen, Christina Wefers, Mark A. J. Gorris, Johannes C. Textor, Leon F. A. G. Massuger, I. Jolanda M. de Vries, Anne M. van Altena

**Affiliations:** ^1^Department of Obstetrics and Gynecology, Radboud University Nijmegen Medical Centre, Nijmegen, Netherlands; ^2^Department of Tumor Immunology, Radboud Institute for Molecular Life Sciences, Radboud University Nijmegen Medical Centre Nijmegen, Netherlands

**Keywords:** regression of ovarian carcinoma, septic peritonitis, immune suppressive tumor environment, anti-tumor immune response, immunotherapy

## Abstract

Despite advances in therapy, ovarian cancer remains the most lethal gynecological malignancy and prognosis has not substantially improved over the past 3 decades. Immunotherapy is a promising new treatment option. However, the immunosuppressive cancer microenvironment must be overcome for immunotherapy to be successful. Here, we present a unique case of spontaneous regression of ovarian carcinoma after septic peritonitis. A 79-year-old woman was diagnosed with stage IIIc ovarian cancer. The omental cake biopsy was complicated by sepsis. Although the patient recovered, her physical condition did not allow further treatment for her ovarian cancer. After 6 months, spontaneous regression of the tumor was observed during surgery. Analysis of the immune infiltrate in the tissues showed a shift from a pro-tumorigenic to an anti-tumorigenic immune response after sepsis. Strong activation of the immune system during sepsis overruled the immunosuppressive tumor microenvironment and allowed for a potent anti-tumor immune response. More understanding of immunological responses in cases with cancer and septic peritonitis might be crucial to identify potential new targets for immunotherapy.

## Highlights

- Ovarian cancer escapes the immune system by inducing an immunosuppressive tumor microenvironment
- Sepsis can induce a potent anti-tumor immune response in ovarian cancer
- The immune system can induce ovarian cancer regression

## Introduction

Ovarian cancer is the most lethal gynecological malignancy. Standard of care for epithelial ovarian cancer patients includes surgical resection and chemotherapy with non-specific cytotoxic drugs. Despite advances in treatment strategies, such as more aggressive surgery, combination chemotherapy, intraperitoneal chemotherapy, hyperthermia and targeted molecular therapy, the 5-year survival rate and quality of life of ovarian cancer patients has not substantially improved over the past 30 years. Therefore, new therapeutic approaches are needed. Immunotherapy and immune modulation are promising treatment options. However, the immunosuppressive ovarian cancer microenvironment must be overcome for successful immunotherapy (1, 2).

We hereby present a unique case of spontaneous regression of a histologically confirmed FIGO stage IIIC serous papillary ovarian carcinoma after a septic peritonitis. The rare phenomenon of spontaneous regression and recovery of cancer after sepsis has been described in several cancer types over the past centuries, including embryonal and breast cancer, renal adenocarcinoma, neuroblastoma, melanoma, and sarcoma or carcinoma of the urinary bladder, but not in ovarian cancer (3). The mechanisms for spontaneous regression of cancer are not clear but the remissions are often associated with concurrent bacterial, fungal, viral or protozoan infections. The most commonly reported symptom wherein spontaneous regression is registered is an acute febrile state that is evoked by either natural or induced acute infection (4).

Septic peritonitis includes inflammation of the peritoneum that can be caused by microorganisms. It is a life-threatening condition that requires hospitalization, resuscitation and close monitoring to prevent systemic spread, which might lead to organ failure. The strong activation of the immune response within the peritoneal cavity during sepsis involves both the innate and adaptive immune system. The host inflammatory response consequent to initial exposure to pathogens is often followed by anti-inflammatory forces and these responses are immunologically highly complex and not completely understood (5–7). Information on septic peritonitis and spontaneous regression of ovarian cancer is even more scarce. More understanding of immunological responses in cases with cancer and septic peritonitis might be crucial to identify potential new targets for immunotherapy in the future.

## Case

A 79-year-old woman with an increasingly distended abdomen, fatigue and dyspnoea was referred to a secondary clinic in the Netherlands. During primary workup with CT-scan, an enlarged ovary and extensive ascites with omental cake were demonstrated. In addition, the serum marker CA-125 was elevated (808 kU/L). Based on histopathological results of an omental biopsy and prior aspiration of ascitic fluid the diagnosis of stage IIIC epithelial ovarian cancer was established. Unfortunately, the biopsy was complicated by a septic peritonitis with fever up to 39.8°C for which she was admitted to the intensive care unit and was treated accordingly. Differential diagnosis involved intra-abdominal contamination or bowel puncture/injury during the biopsy procedure. Although the patient recovered, her physical condition afterwards did not allow a surgical debulking procedure or neoadjuvant chemotherapy. She was discharged from the hospital with palliative comfort care.

Six months later, she was referred to our hospital for a second opinion as she was in a good physical and mental condition. During physical examination she did not show signs of lymphadenopathy, ascites or an abdominal mass. The serum marker CA-125 was normal (10 E/mL). An additional CT-scan demonstrated no pulmonary or pleural abnormalities and no signs of lymphadenopathy. Both the left ovary (42 × 24 mm) and the right ovary (23 × 11 mm) were slightly enlarged. There were no signs of free fluid, ascites, omental cake, peritonitis carcinomatosis, or other abnormalities.

An uncomplicated laparoscopic bilateral salpingo-oophorectomy was performed including peritoneal biopsies and a partial omentectomy along with free fluid collection from the pouch of Douglas. Intraoperative findings showed an enlarged left ovary, without further residual tumor deposits intra-abdominally. In concordance with the prior omental biopsy, a high-grade serous carcinoma was noted within the left ovary. There were no tumor deposits detected in the right ovary, the omentum or in any of the other biopsies. After counseling, the patient opted for expectant management and did not receive adjuvant systemic chemotherapy. To date, 42 months after diagnosis, she shows no signs of recurrent or progressive disease with a serum CA-125 at 11.0 E/mL.

We conclude that this patient represents a very rare case, and the only case described in literature, of spontaneous regression of a histologically confirmed stage IIIC ovarian carcinoma after a septic peritonitis. Since we were interested in the immunological mechanisms at play in our patient, we set out to study this further.

## Materials and methods

### Institutional and ethical approval

This study was carried out in accordance with the recommendation of the “Code of conduct for responsible use of human tissue,” established by the Federa (Federation of Dutch Medical Scientific Societies). The study was officially deemed exempt from medical ethical approval. A signed consent form was obtained from the patient described in this case report. Anonymous rest material was used as control tissue. The experiments were carried out in accordance with the guidelines and regulations of the Radboudumc. Criteria for ovarian cancer regression were based on clinical data, such as normalization of CA-125 serum levels, normal physical examination, no abnormalities on imaging (ultrasound/CT/MRI) and tumor-free biopsies evaluated by a pathologist.

### Multiplex immunohistochemistry

Multiplex immunohistochemistry was performed using sequential staining cycles as described elsewhere (8). In brief, tissue sections of 4 μm were cut form FFPE tissue. Sections were deparaffinized in xylene, rehydrated and washed in tap water. Sections were stained with a T cell panel and macrophage panel. T cell panel: CD3 (RM-9107, Thermo Fisher), CD8 (M7103, Dako), Foxp3 (14-4777, eBioscience Affymetrix). Macrophage panel: CD68 (M087601, Dako), CD163 (CD163-L-CE, Leica). Both panels contained CKAE1/AE3 (ab86734, Abcam) to visualize tumor cells. Heat mediated antigen retrieval was performed in citrate. Protein block was performed with TBS-Tween containing 1% BSA. Primary antibodies were incubated for 1 h at room temperature. Sections were incubated with BrightVision poly-HRP-anti-MS/Rb/Rt IgG (DPVO999HRP, ImmunoLogic) for 30 min, followed by visualization with the Opal color IHC kit (NEL801001KT), (PerkinElmer). A second antigen retrieval step with citrate or Tris-EDTA was performed to remove the antibody-TSA complex and to continue with the next staining cycle. Tissue sections were counterstained with DAPI and mounted in Fluoromount-G (0100-01; SouthernBiotech).

### Imaging and analysis

Tissue slides were imaged with the PerkinElmer Vectra system (Vectra 3.0.3, Perkin Elmer). InForm software (Version 2.2.1, PerkinElmer) was used for image analysis. Spectral libraries were built from single stains and used to unmix multispectral images.

## Results

**Figure 1:**
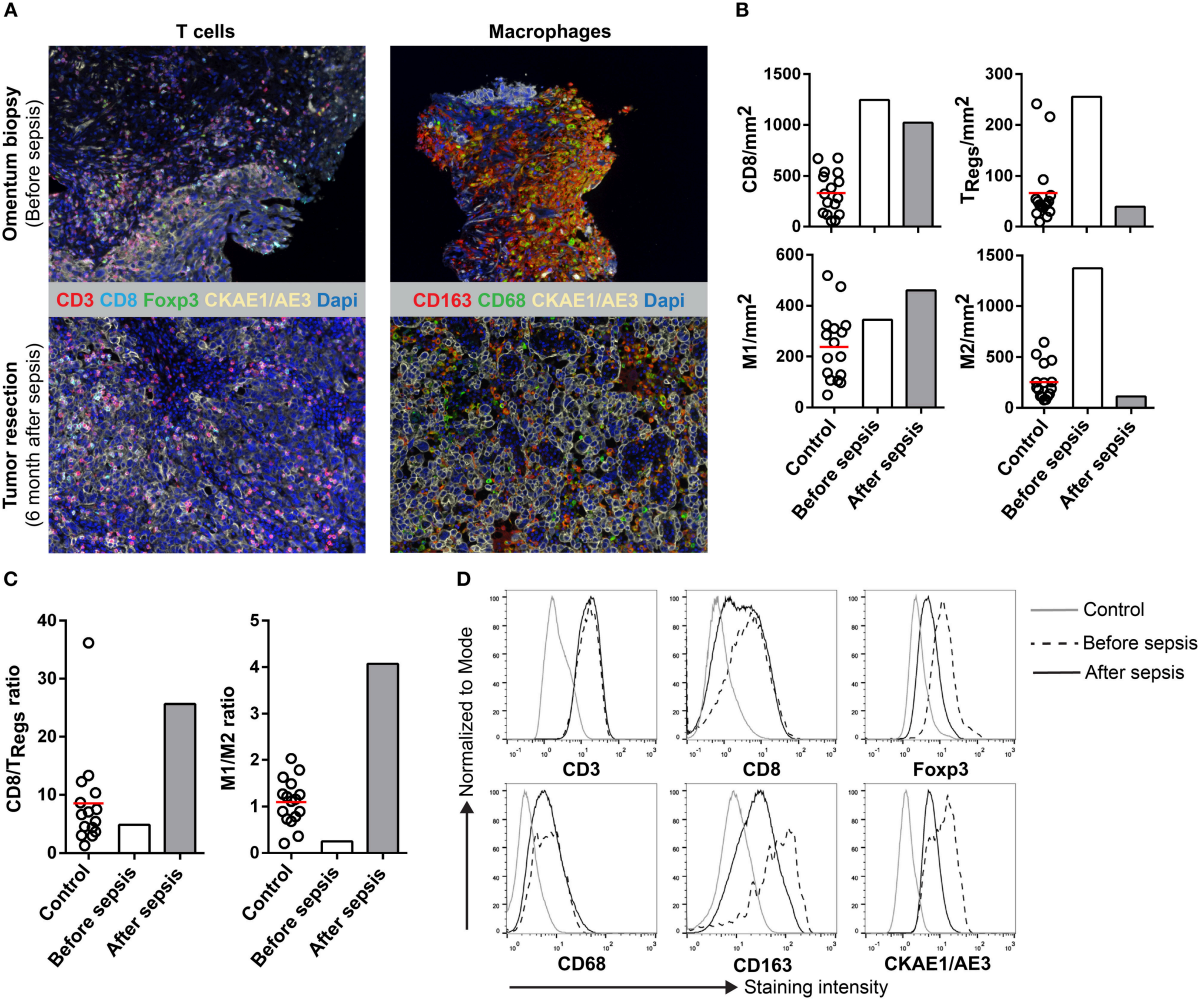
Multiplex IHC on tissue before and after sepsis. **(A)** Images showing T cell infiltration and macrophage infiltration in tissue before (omentum biopsy) and after (tumor resection) sepsis. CKAE1/AE3 was used to visualize tumor cells. Effector T cells were characterized as CD3^+^CD8^+^, regulatory T cells as CD3^+^Foxp3^+^, M1 macrophages as CD68^+^, and M2 macrophages as CD163^+^. **(B)** CD8 T cells, T_Regs_, M1 and M2 macrophages per mm^2^. Immune cells were quantified in control tissue, and in tissue obtained before and after sepsis. **(C)** CD8 effector T cell/T_Reg_ ratio and M1/M2 ratio before and after sepsis. **(D)** Average staining intensity of immune cell markers before and after sepsis. Unstained tissue was used as control. Untreated primary tumor from stage III and IV ovarian cancer patient were used as control (*n* = 16). Red line indicates mean.

Multiplex immunohistochemistry was used to visualize the tumor-infiltrating lymphocytes (CD3, CD8, Foxp3) (9) and macrophages (CD68, CD163) (10) and to quantify the infiltrating immune cells in the omental cake biopsy taken before sepsis and in the carcinoma resected 6 month after sepsis. Untreated primary tumors from stage III and IV high-grade serous ovarian cancer patients that did not suffer from sepsis were used as a control. Sections were stained using an automated-imaging system and the cell ratios were calculated. The biopsy (before sepsis) was highly infiltrated by effector CD8^+^ T cells, as well as Foxp3^+^ regulatory T cells (T_Regs_) (Figures 1A,B). In contrast, the number of infiltrating T_Regs_ was decreased in the carcinoma (after sepsis). Overall, there was a shift in the CD8/T_Reg_ ratio (Figure 1C), which was accompanied by a decrease in the Foxp3 staining intensity (Figure 1D). Before the sepsis, the ratio was comparable to the mean of the control tumors. After the sepsis, the CD8/T_Reg_ ratio increased, indicating that the carcinoma contained relatively more effector CD8^+^ T cells compared to T_Regs_. The biopsy and carcinoma were also stained for macrophage markers. Macrophages can acquire a M1 (CD68^+^CD163^−^) or M2 (CD68^−/+^CD163^+^) phenotype, having anti- or pro-tumorigenic effects, respectively (11). Whereas the biopsy contained high numbers of M1 and M2 macrophages, the presence of M2 macrophages was markedly decreased after sepsis in the carcinoma (Figures 1A,B). Before sepsis the M1/M2 ratio was at the lower end of the spectrum of control tumors, whereas after the sepsis it was higher than the ratio of control tumors (Figure 1C). The staining intensity for CD163^+^ M2 macrophages was decreased after the sepsis (Figure 1D). These results point toward a decrease of immunosuppressive cells in the tumor microenvironment after sepsis.

## Discussion

More than 60–80% of ovarian cancer patients are diagnosed at an advanced stage due to the lack of symptoms and adequate screening methods. The 5-year survival rates in stage III and IV ovarian cancer are ~35 and ~15%, respectively. Ovarian cancer is an intra-peritoneal disease, which tends not to metastasize outside the peritoneal cavity. Current treatment for advanced disease consists of cytoreductive surgery combined with chemotherapy. Despite the good initial response to primary therapy around 70–85% of the patients with advanced stage of disease develop recurrence and eventually die.

Research has shown that the immune system plays an important role in ovarian cancer progression and is able to influence clinical outcome. Activation of the immune system requires presentation of antigen by antigen-presenting cells (APCs) to T cells. The cytotoxic effects are driven by a combination of T-lymphocyte activity, antibody-dependent mechanisms, and natural killer (NK) cell activation. Most tumors are in some way recognized by the immune system as tumor infiltrating lymphocytes (TILs) have been observed within and around the tumor tissue in a variety of different cancers. In ovarian cancer, a high number of TILs in the tumor are associated with a favorable clinical outcome (1).

However, ovarian cancer can escape the immune system via various mechanisms: (1) evasion of immune recognition; (2) secretion of immune suppressive factors; and (3) recruitment of immune suppressive cells (1, 12). T_Regs_ and M2 macrophages in the ovarian cancer microenvironment are able to inhibit the anti-tumor immune response of cytotoxic T cells, either by secretion of immunosuppressive cytokines like IL-10 and TGF-β or via a cell-cell contact-dependent mechanism (12). Accumulation of T_regs_ at the tumor site is associated with reduced survival of ovarian cancer patients (1). Immunotherapy needs to overcome the immunosuppressive tumor microenvironment to evoke an effective anti-tumor immune response.

Different immunotherapeutic strategies can be used to boost the anti-tumor immune response, including vaccination with tumor-specific dendritic cells, adoptive T cell therapy and immune checkpoint blockade (13–16). Several clinical trials are currently investigating the safety and feasibility of these approaches in ovarian cancer. Two phase 1 studies showed that DC vaccination (using whole-tumor lysate) alone or in combination with bevacizumab or cyclophosphamide was safe, well tolerable and effective in eliciting a broad anti-tumor immune response in patients with recurrent ovarian, fallopian tube or peritoneal cancer (17, 18). Immunotherapy using *ex-vivo* expanded tumor-infiltrating lymphocytes or genetically engineered T cells also demonstrated feasibility and safety in platinum-resistant or recurrent ovarian cancer with manageable toxicities (19–21).

The inhibition of immune checkpoints has shown great success in the treatment of melanoma patients and, therefore, data on ovarian cancer are eagerly awaited. Antibodies against CTLA-4 (ipilumumab), PD-1 (nivolumab, pembrolizumab), and PD-L1 (avelumab, durvalumab) are currently tested in clinical trials and seem to be well tolerated by ovarian cancer patients (22, 23). For example, a phase II study for nivolumab in patients with platinum-resistant ovarian cancer showed partial response and stable disease in 20 and 25% of the patients, respectively. Their overall disease control rate in 20 patients was 45% with grade 3 or 4 treatment-related adverse events occurring in eight (40%) patients (24). Others showed that pembrolizumab and avelumab in phase 1 clinical trials were very well tolerated, with an acceptable safety profile and with durable antitumor activity in patients with advanced ovarian, fallopian tube or peritoneal cancer. Regarding treatment-related adverse events after treatment with pembrolizumab, 73.1% of the patients had grade 1–2 adverse events with only one grade 3 adverse event (23). For avelumab, grade 3 or 4 treatment-related adverse events occurred in 10.2% (25–27). Currently there are two other phase 2 clinical trials underway to determine the safety, feasibility and efficacy of durvalumab (anti-PD-L1) and ipilimumab (anti-CTLA-4) in first-line neo-adjuvant setting for ovarian cancer (combined with chemotherapy), or as monotherapy in recurrent platinum sensitive ovarian cancer, respectively. These results are expected in 2019 and 2021.

Although these data might seem promising, many of the clinical trials are ongoing and progress in the development of these highly specific immunotherapeutic approaches to ovarian cancer treatment is slow and a crucial clinical break-through is still missing.

Sepsis induces a hyperactive immune system in response to invading pathogens (28). Immune cells are activated by the recognition of pathogen-associated molecular patterns (PAMPs) that are present on microorganisms (29). This group of various pathogens, which among others include lipopolysaccharides (LPS), a component of bacterial cell walls, interact with Toll-like receptors (TLRs) on various immune cells (T lymphocytes, DCs and neutrophils). Binding of the TLRs by PAMPs induces activation of dendritic cells and cytotoxic T cells, leading to a highly improved proinflammatory response that is able to clear the invading pathogens. Sepsis not only stimulates the adaptive immune response but it also activates innate immune cells such as, granulocytes and macrophages. We detected a shift in the CD8^+^ effector T cells/T_Reg_ ratio and M1/M2 ratio when comparing the immune cell infiltrate before and after sepsis. These results indicate that sepsis is able to overrule the immunosuppressive tumor microenvironment, inducing a long-lasting anti-tumor immune response that is still detectable 6 months after the sepsis. Non-specific activation (e.g., natural or induced acute infection) of the innate immune system may critically support the initiation of a functional specific immune response against cancer.

The hyperactive immune response is accompanied by fever. A febrile state enhances the cytokine secretion of immune cells with an increase in proinflammatory cytokines. Furthermore, cancer cells are more fragile and vulnerable to heat with apoptosis taking place at lower temperatures compared to normal cells. Necrotic or heat-stressed cancer cells can function as antigens or PAMPs and thereby generate an immune-stimulating environment (30). Recently, in patients with metastatic melanoma treated with immunotherapy, fever of 39.5°C or above was an independent factor for improved survival and objective tumor response (31).

The use of bacteria, fungi, or components thereof might be a promising path for non-specific immunotherapy against tumors and might be a helpful adjuvant for specific immunotherapy. The choice of pathogen or PAMP is essential in this scenario. For example, activation of TLR-4 by LPS or its derivates drives macrophage polarization toward an M1 pro-inflammatory phenotype (32). Hence, TLR-4 agonists could be used to target tumor-associated macrophages that counteract anti-tumor immunity. However, since *in vitro* studies have shown that TLR-4 on ovarian cancer cells promotes cell proliferation and survival, more research is needed to investigate the use of TLR-4 agonists (33, 34). The severity of the hyperactive immune response is amongst others determined by pathogen-load and virulence factor. If the activation of the immune system is too strong, organ failure and death may occur. On the other hand, if activation of the immune system is not strong enough, the immunosuppressive tumor microenvironment may not be overcome (35, 36). In addition to modern antigen-specific antibody- and vaccine-based immunologic cancer therapies, non-specific immunotherapies might serve as an ideal tool. More insight in the interaction between ovarian cancer and the immune system is needed to develop effective anti-tumor immunotherapy. We believe that non-specific activation of a broad immune response could tilt the balance from an immune suppressive to an immune active environment. This could have an impact on ovarian cancer treatment. Can we boost the immune system using bacterial or fungal components to evoke a potent and long-lasting anti-tumor immune response?

## Author contributions

TR, CW, LM, IdV, and AvA were responsible for the study concept and design. TR, CW, and AvA drafted the report. TR and CW were responsible for data collection. CW, MG, and JT performed image analysis. CW and IdV did the statistical analyses. TR, CW, LM, IdV, and AvA were involved with administrative, technical, or material support. All authors were responsible for the integrity and accuracy of the data and approved the final version of this report.

### Conflict of interest statement

The authors declare that the research was conducted in the absence of any commercial or financial relationships that could be construed as a potential conflict of interest.
